# Clinical aspects and self-reported symptoms of sequelae of *Yersinia enterocolitica* infections in a population-based study, Germany 2009–2010

**DOI:** 10.1186/1471-2334-13-236

**Published:** 2013-05-23

**Authors:** Bettina M Rosner, Dirk Werber, Michael Höhle, Klaus Stark

**Affiliations:** 1 Robert Koch Institute, Department of Infectious Disease Epidemiology, DGZ-Ring 1, Berlin, 13086, Germany

**Keywords:** *Yersinia enterocolitica*, Sequelae, Reactive arthritis, Erythema nodosum

## Abstract

**Background:**

Foodborne *Yersinia enterocolitica* infections continue to be a public health problem in many countries. Consumption of raw or undercooked pork is the main risk factor for yersiniosis in Germany. Small children are most frequently affected by yersiniosis. In older children and young adults, symptoms of disease may resemble those of appendicitis and may lead to hospitalization and potentially unnecessary appendectomies. *Y. enterocolitica* infections may also cause sequelae such as reactive arthritis (ReA), erythema nodosum (EN), and conjunctivitis.

**Methods:**

We studied clinical aspects of yersiniosis, antimicrobial use, and self-reported occurrence of appendectomies, reactive arthritis, erythema nodosum and conjunctivitis. To assess post-infectious sequelae participants of a large population-based case–control study on laboratory-confirmed *Y. enterocolitica* infections conducted in Germany in 2009–2010 were followed for 4 weeks.

**Results:**

Diarrhea occurred most frequently in children ≤4 years (95%); abdominal pain in the lower right quadrant was most common in children 5–14 years of age (63%). Twenty-seven per cent of patients were hospitalized, 37% were treated with antimicrobials. In 6% of yersiniosis patients ≥5 years of age, appendectomies were performed. Self-reported symptoms consistent with ReA were reported by 12% of yersiniosis patients compared to 5% in a reference group not exposed to yersiniosis. Symptoms consistent with EN were reported by 3% of yersiniosis patients compared to 0.1% in the reference group. Symptoms of conjunctivitis occurred with the same frequency in yersiniosis patients and the reference group.

**Conclusions:**

Acute *Y. enterocolitica* infections cause considerable burden of illness with symptoms lasting for about 10 days and hospitalizations in more than a quarter of patients. The proportion of yersiniosis patients treated with antimicrobial drugs appears to be relatively high despite guidelines recommending their use only in severe cases. Appendectomies and post-infectious complications (ReA and EN) are more frequently reported in yersiniosis patients than in the reference group suggesting that they can be attributed to infections with *Y. enterocolitica*. Physicians should keep recent *Y. enterocolitica* infection in mind in patients with symptoms resembling appendicitis as well as in patients with symptoms of unclear arthritis.

## Background

Yersiniosis caused by *Yersinia enterocolitica* infection is a foodborne enteric illness often related to consumption of raw or undercooked pork [1-3]. *Y. enterocolitica* infections are notifiable in Germany and most other European countries and are being monitored by the foodborne diseases active surveillance network (FoodNet) in the United States. In 2011, about 3,400 *Y. enterocolitica* infections were notified to the Robert Koch Institute (RKI), the federal public health agency in Germany. This corresponds to an incidence of 4.2 *Y. enterocolitica* infections/100,000 population. The incidence of yersiniosis in Germany is higher than in most other European countries and the United States. Young children, in particular one-year-olds, are most frequently affected by yersiniosis [4]. Symptoms of yersiniosis include diarrhea and abdominal pain. In older children and young adults symptoms may resemble those of appendicitis (pseudoappendicitis). In some cases, *Yersinia* infections may lead to extraintestinal complications such as reactive arthritis (ReA), erythema nodosum (EN), and conjunctivitis [5,6]. Furthermore, chronic gastrointestinal disorders such as irritable bowel syndrome, functional constipation, and gastroesophageal reflux disease have also been reported as being associated with preceding *Y. enterocolitica* infections [7]. Investigations on the occurrence of sequelae of *Yersinia* infections, in particular ReA and EN, are often related to outbreaks with recent investigations focusing on *Y. pseudotuberculosis* rather than *Y. enterocolitica* outbreaks [8-10]. There is a strong need for population-based studies that estimate the incidence of sequelae following sporadic *Yersinia*, in particular *Y. enterocolitica* infections [11-14]. In 2009–2010, we conducted a large case–control study to determine risk factors for sporadic *Y. enterocolitica* infections [3]. As part of this study, we assessed clinical characteristics, antimicrobial use, as well as the frequency and determinants of appendectomies associated with acute *Y. enterocolitica* infections. In addition, we studied the occurrence of self-reported post-infectious symptoms consistent with ReA, EN, and conjunctivitis in yersiniosis patients and a population-based reference group with a follow-up period of about one month.

## Methods

### Study design

Symptoms of yersiniosis and, for comparison, gastrointestinal symptoms in a population-based reference group, were assessed in participants of a large case–control study on risk factors for yersiniosis conducted in 2009–2010 in 5 federal states in Germany [3]. The methodology of the original case–control study has been previously described in detail [3]. Briefly, a case was defined as a *Y. enterocolitica* infection culture-confirmed from stool in a person with at least one symptom of an acute gastrointestinal illness (diarrhea, abdominal pain, tenesma, vomiting, fever) notified to the local health department during the study period (15 April 2009–30 June 2010). For the case–control study, a reference group (controls) was randomly selected from population registries and frequency matched to yersiniosis case-patients by age group (0–4 years, 5–14 years, ≥15 years) and federal state (ratio 3 controls per 1 case). Parents or caregivers were asked to complete the questionnaire for children <15 years of age. The federal commissioner of data protection and freedom of information and the state data protection commissioners of the federal states participating in the study had approved data collection and storage procedures. The ethics committee of Charité, University Medicine, Berlin, had no ethical concerns regarding the study.

In addition to assessing the frequency of gastrointestinal symptoms in yersiniosis patients and the reference group, we conducted two cohort studies. In cohort study 1, we used case-patients of the original case–control study to investigate the association of symptoms of acute yersiniosis with the outcomes hospitalization and appendectomy, respectively. In cohort study 2, we studied the incidence of possible sequelae of yersiniosis (ReA, EN, conjunctivitis). To investigate the association of a preceding *Y. enterocolitica* infection with symptoms of possible sequelae, yersiniosis case-patients and, as a reference group, the population-based controls were enrolled as a cohort in a follow-up questionnaire-study. Here, *Y. enterocolitica* infection was considered the exposure and self-reported symptoms of possible sequelae were the outcomes. In contrast to the original case–control study where cases and controls had been frequency-matched by age group and federal states, the exposed and the unexposed group were no longer frequency-matched. An unexposed reference group was used because in our study we assessed self-reported symptoms of sequelae, that is, without clinical confirmation. The inclusion of a reference group allowed us to estimate the background rate of self-reported “sequelae-like” symptoms unrelated to a preceding *Y. enterocolitica* infection.

Severity of gastrointestinal illness in yersiniosis patients was defined based on self-reported answers. We applied 3 different criteria for severity: 1) duration of gastrointestinal symptoms longer than the median duration (>10 days); 2) hospitalization due to acute gastrointestinal symptoms; 3) similar to Schiellerup et al. [15], three severity levels were formed: mild: diarrhea and/or abdominal pain and/or tenesma and/or vomiting and/or other symptoms, e.g., headache; moderate: symptoms of mild severity plus fever and/or abdominal pain in lower right quadrant; severe: at least one symptom plus visible blood in stool and/or hospitalization.

### Data collection

#### Description of clinical aspects of yersiniosis and cohort study 1

Data on symptoms and duration of yersiniosis, date of disease onset, hospitalization, antimicrobial treatment, appendectomy associated with *Y. enterocolitica* infection, absence from work, and reasons for contacting a health care provider during acute yersiniosis was collected from yersiniosis patients of the original case–control study on risk factors for yersiniosis using a self-administered questionnaire [3]. The population-based reference group was asked about gastrointestinal symptoms in the 7 days before completing the self-administered questionnaire and whether they had consulted a physician because of these symptoms. The assessment of the frequency of appendectomies in the reference group referred to the 4 weeks before completing the questionnaire.

#### Cohort study 2 (possible sequelae of yersiniosis)

Participants of the original case–control study had been asked to indicate in writing whether they agreed to further interviewing by RKI staff and to provide contact data. Those who consented were interviewed by telephone with a standard questionnaire about 4 weeks after onset of yersiniosis (case-patients) or completing the self-administered questionnaire (reference group). The questionnaire used for the telephone interviews queried about new onset of symptoms consistent with ReA, EN, and conjunctivitis, and consultation of a physician because of these symptoms. Symptoms were considered as probable indications of sequelae in yersiniosis patients if they started at least one day, but no longer than 36 days after onset of gastrointestinal symptoms and lasted for 3 or more days. The following symptoms were considered as consistent with ReA: joint pain, joint swelling, morning joint or back stiffness lasting for more than an hour, low back pain, buttocks pain, and heel pain. A case of probable ReA was defined as the new onset of at least one of these symptoms. Probable EN was defined as the new occurrence of tender reddish-violet or yellowish-greenish subcutaneous spots or nodules, in particular on the lower extremities or arms. Probable conjunctivitis/iritis was defined as new occurrence of redness and/or swelling of conjunctiva or iris.

### Data entry and analysis

Data was entered into an EpiData database (version 3.1; EpiData Association, Odense, Denmark) and analyzed with Stata 12 (Stata Corporation, College Station, TX, USA). For cohort study 1 and 2, risk ratios (RR) and 95% confidence intervals (CI) were estimated using binomial regression models with log-link function (log-binomial model) adjusted for age group and sex. This method is one recommended modeling approach to obtain RRs in models where the response is binomial [16,17]. Statistical significance was assessed by Wald test. *P* values <0.05 were considered statistically significant.

## Results

### Study population

In the original case–control study, 571 cases with a median age of 8 years (interquartile range (IQR) 2–17 years; 56% male) and 1,798 controls with a median age of 9 years (IQR 3–26 years; 49% male) had been included [3]. Three hundred and fifty-one yersiniosis case-patients (62%) and 819 persons of the control group (46%) were interviewed regarding the new onset of symptoms consistent with probable sequelae or “sequelae-like” symptoms, respectively, in cohort study 2. The median time interval between onset of symptoms of yersiniosis and the telephone interview was 35 days (IQR 26–71 days). The reference group was interviewed on average (median) 33 days after completing the initial study questionnaire (IQR 25–90 days).

### Description of clinical aspects of *Y. enterocolitica* infection and cohort study 1

Patients most frequently reported diarrhea and abdominal pain as symptoms of *Y. enterocolitica* infection (Table 1). In 95% of children <5 years diarrhea was reported. The frequency of reported pain in the lower right abdomen was highest in children 5–14 years of age (Table 1). Median duration of symptoms was 10 days (Table 2). The main reasons for consulting a physician were severe abdominal pain (62%), diarrhea on 3 or more consecutive days (52%), and severe diarrhea (45%). Severe diarrhea or diarrhea on 3 or more consecutive days were the most frequently named reasons for seeking healthcare in children <5 years of age (72% and 68%, respectively), severe abdominal pain was the most frequently named reason in persons ≥5 years of age (78%). Diarrhea, abdominal pain, tenesma, and fever were more frequent in yersiniosis patients positive for serotype O:3 compared with non-O:3 serotypes (i.e., O:9, O:5,27, or non-specified serotype). Yersiniosis patients positive for serotype O:3 (n=481) were significantly younger than patients positive for other non-O:3 serotypes (n=33) (median age 7 years (IQR 2–15 years) versus 11 years (IQR 8–45 years)).

**Table 1:** Comparison of self-reported gastrointestinal symptoms of yersiniosis patients and persons without yersiniosis (reference group)

	**Yersiniosis patients**				**Reference group**			
**Symptom**	**All (N=571) n (%)**	**0-4 years (n=214) n (%)**	**5-14 years (n=197) n (%)**	**≥15 years (n=160) n (%)**	**All (N=1,798) n (%)**	**0-4 years (n=592) n (%)**	**5-14 years (n=668) n (%)**	**≥15 years (n=537) n (%)**
Diarrhea	460 (80.6)	203 (94.9)	131 (66.5)	126 (78.8)	96 (5.4)	33 (5.6)	25 (3.8)	38 (7.2)
Abdominal pain	419 (77.2)	115 (60.5)	168 (87.1)	136 (85.0)	118 (6.7)	28 (4.9)	41 (6.2)	49 (9.4)
Pain in lower right abdomen	203 (42.0)	21 (14.1)	118 (63.4)	64 (43.2)	21 (1.2)	2 (0.4)	4 (0.6)	15 (2.9)
Tenesma	106 (20.5)	36 (20.1)	35 (18.7)	35 (23.0)	47 (2.7)	17 (3.0)	8 (1.2)	22 (4.2)
Fever	299 (54.8)	139 (65.0)	124 (63.3)	36 (26.5)	97 (5.5)	64 (10.9)	27 (4.1)	6 (1.2)
Vomiting	110 (19.4)	43 (20.4)	47 (23.9)	20 (12.5)	67 (3.8)	36 (6.1)	23 (3.5)	8 (1.5)
Visible blood in stool	31 (5.5)	19 (9.1)	3 (1.6)	9 (5.7)	9 (0.5)	2 (0.3)	3 (0.5)	4 (0.8)
Other symptoms	354^a^ (63.3)	111 (53.1)	141 (72.7)	102 (65.4)	222 (13.5)	101 (18.2)	59 (9.8)	60 (12.7)

^a^175 case-patients provided details on other symptoms. Most frequently named other symptoms were headache (n=54), fatigue (n=28), and nausea (n=24).

Symptoms in the population-based reference group occurred within the 7 days preceding completing the questionnaire of a case–control study, Germany 2009–2010.

**Table 2:** Clinical aspects of infections in different age groups of yersiniosis patients, Germany 2009–2010 (N=571)

**Burden**	**All**	**0-4 years**	**5-14 years**	**≥15 years**
Duration of symptoms (median)	10 days (IQR^a^ 6–14 days)	13 days (IQR 9–14 days)	7 days (IQR 4–14 days)	11 days (IQR 7–16 days)
Hospitalization (n)	27% (153)	13% (28)	46% (89)	23% (36)
Duration of hospital stay (median)	4 days (IQR 3–6 days)	5 days (IQR 3–7 days)	4 days (IQR 3–5 days)	5 days (IQR 3–7 days)
Appendectomy (n)	4% (22)	0% (0)	6% (12)	6% (10)
Treatment with antimicrobial drugs (n)	37% (199)	30% (61)	26% (48)	60% (90)
Absence from work^b^ (n)	46% (204)	40% (67)	35% (58)	71% (79)
Duration of absence from work (median)	5 days (IQR 3–9.5 days)	5 days (IQR 3–10 days)	4 days (IQR 2–7 days)	6 days (IQR 4–10 days)

^a^interquartile range.

^b^parents of sick children or case-patients of working age (15–64 years) themselves.

#### Hospitalization

Overall, 27% of patients reported hospitalization due to *Y. enterocolitica* infection. The proportion of hospitalized patients was highest in children 5–14 years of age (Table 2). Hospitalization was significantly associated with pain in the lower right abdomen (Table 3). The median duration of hospital stay was 4 days (Table 2).

**Table 3:** **Risk ratio of hospitalization and appendectomy by symptom of *Y. enterocolitica *infection in yersiniosis patients, Germany 2009–2010 (N=571)**

	**Hospitalization**		**Appendectomy**	
**Symptom**	**Risk ratio**^**a **^**(95% CI**^**b**^**)**	***P *value**^**c**^	**Risk ratio**^**a **^**(95% CI)**	***P *value**^**c**^
Diarrhea	0.5 (0.4 -0.7)	<0.001	0.3 (0.1-0.6)	0.001
Abdominal pain	0.9 (0.6 -1.2)	0.406	0.4 (0.2 -1.0)	0.049
Pain in lower right abdomen	2.7 (1.9-3.8)	<0.001	19.0 (2.6-140.7)	0.004
Tenesma	0.9 (0.6-1.2)	0.428	0.6 (0.2-2.1)	0.451
Fever	2.0 (1.5-2.8)	<0.001	2.3 (0.9 -5.6)	0.075
Vomiting	1.7 (1.3 -2.1 )	<0.001	1.0 (0.3-2.8)	0.929
Visible blood in stool	0.9 (0.5 -1.9)	0.871	.^d^	0.626
Other symptoms	1.2 (0.9-1.7)	0.154	0.6 (0.3 -1.4)	0.277

^a^adjusted for age group and sex in a log-binomial regression model.

^b^Confidence interval.

^c^Wald test.

^d^No RR estimate was calculated because none of the 22 patients with appendectomy had reported visible blood in stool.

#### Appendectomies

Appendectomies triggered by symptoms of *Y. enterocolitica* infection were reported by 22/571 case-patients (4%). The median age of yersiniosis patients with appendectomy was higher (11.5 years, IQR 9–18 years) than the median age of patients without appendectomy (8 years, IQR 2–17 years). Appendectomy was strongly and significantly associated with pain in the lower right abdomen (Table 3). Of note, only one person out of 1,798 persons in the reference group (0.06%) reported an appendectomy in the 4 weeks before completing the self-administered questionnaire. Thus, yersiniosis patients were more than 70 times more likely to report an appendectomy than the reference group not exposed to yersiniosis (RR 73.5; 95% CI: 9.9 – 544.0; *P*<0.001).

#### Absence from work

In total, 46% of respondents of working age were absent from work either because their children suffered from yersiniosis (38%) or because they suffered from yersiniosis themselves (71%). The median time period of absence from work was 5 days (IQR 3–9.5 days) (Table 2).

#### Antimicrobial treatment

In 37% of case-patients, the infection was treated with antimicrobial drugs. This proportion was higher (60%) in persons ≥15 years of age than in children (Table 2). Antibiotic treatment was not significantly associated with any of the symptoms queried in the questionnaire. Median duration of gastrointestinal illness was 12 days in case-patients who reported antimicrobial treatment and 10 days in case-patients who did not receive antimicrobial drugs. There was no significant association of antimicrobial treatment with a presumed severe course of illness as reflected by long duration (>10 days) of gastrointestinal symptoms (RR 1.1; 95% CI: 0.9-1.4; *P*=0.200), hospitalization (RR 0.9; 95% CI: 0.6-1.2; *P*=0.371), or symptoms considered as severe according to the criteria defined above (severe vs. moderate or mild, RR 1.0; 95% CI: 0.7-1.2; *P*=0.775).

#### Gastrointestinal symptoms in the reference group

The majority of the reference group (68%) did not have any symptoms in the 7 days before completing the self-administered questionnaire. Seven per cent of persons in the reference group reported abdominal pain and 5% reported diarrhea (Table 1). The frequency of consultation of a physician varied with age group (0–4 years: 40% (n=63); 5–14 years: 34% (n=37), ≥15 years: 23% (n=25)) and symptom (fever: 59% (n=56); visible blood in stool 56% (n=5); vomiting 33% (n=24); diarrhea 27% (n=25); tenesma 26% (n=12); abdominal pain 25% (n=29); pain in lower right abdomen 24% (n=5); other symptoms 41% (n=88)). A stool sample was taken from 6/25 persons (24%) with diarrhea who sought medical care.

### Cohort study 2 (post-infectious symptoms of ReA, EN, conjunctivitis)

About 12% of yersiniosis patients (n=41) reported at least one symptom consistent with probable ReA (Table 4). The most frequently reported symptom was joint pain (6%; n=20), mainly in the knees (n=14), elbows (n=9), and ankles (n=5). Seven yersiniosis patients (17%) reported two symptoms and 4 patients (10%) reported more than two symptoms of ReA. The incidence of symptoms of probable ReA increased with age. About 25% of case-patients ≥15 years of age (n=23) were affected by symptoms of probable ReA versus 3% of children aged <5 years (n=4) and 12% of children aged 5–14 years (n=14). The median age of yersiniosis patients with symptoms of probable ReA was 18 years (IQR 10–34 years), case-patients without these symptoms were significantly younger (median age 7 years, IQR 2–14 years; *P*<0.001). Eleven yersiniosis patients (3%; median age 10 years; IQR 2–20 years) reported symptoms consistent with EN. Sixteen yersiniosis patients (5%; median age 5 years; IQR 2–16.5 years) reported symptoms consistent with conjunctivitis (Table 4). The median age of case-patients with symptoms of probable EN or conjunctivitis was not significantly different from the age of case-patients without these symptoms. The occurrence of symptoms of probable sequelae was not associated with gender. Symptoms of ReA were not associated with *Y. enterocolitica* serotype (O:3 vs. non-O:3). We found a statistically significant negative association of serotype O:3 with occurrence of EN (RR 0.2, 95% CI 0.1-0.9; *P*=0.028). A link of conjunctivitis and serotype was not assessed because none of the case-patients positive for non-O:3 serotypes reported conjunctivitis symptoms.

**Table 4:** **Symptoms consistent with sequelae of *Y. enterocolitica *infection (reactive arthritis, erythema nodosum, conjunctivitis) in yersiniosis patients (N=351) and “sequelae-like” symptoms in a population-based reference group (N=819), Germany 2009-2010**

**Symptom**	**Yersiniosis patients n (%)**	**Reference group n (%)**	**Risk ratio**^**a **^**(95% confidence interval)**	***P *value (Wald test)**
Any symptom of sequelae	62 (19.1)	67 (8.3)	2.3 (1.7 3.2)	<0.001
Any symptom of reactive arthritis	41 (12.4)	39 (4.8)	2.6 (1.7 -3.9)	<0.001
Joint pain	20 (5.9)	19 (2.3)		
Joint swelling	9 (2.6)	1 (0.1)		
Lower back pain	15 (4.5)	11 (1.4)		
Buttocks pain	8 (2.4)	2 (0.3)		
Heel pain	7 (2.1)	11 (1.4)		
Morning joint or back stiffness lasting >1 hour	4 (1.2)	3 (0.4)		
Erythema nodosum	11 (3.2)	1 (0.1)	25.1 (3.3-194.7)	0.002
Conjunctivitis/iritis	16 (4.6)	29 (3.6)	1.2 (0.7-2.3)	0.465

^a^adjusted for age group and sex in a log-binomial regression model.

Yersiniosis patients were about 3-times more likely to report at least one symptom of ReA and about 25-times more likely to report symptoms of EN compared to persons without yersiniosis (Table 4). Yersiniosis was not associated with symptoms of conjunctivitis (Table 4). The median time interval between onset of symptoms of yersiniosis and first onset of symptoms consistent with sequelae was 15 days for ReA (IQR 7–28 days), 10 days for EN (IQR 5–14 days), and 23 days for conjunctivitis (IQR 20–27 days). We did not find an association of severity of *Y. enterocolitica* infection and new onset of symptoms consistent with sequelae regardless of the definition of severity that was applied. Median duration of gastrointestinal symptoms was 13 days (IQR 6–14 days) for yersiniosis patients who developed symptoms of ReA, 8 days (IQR 4–14 days) for patients who developed EN, and 10 days (IQR 6–14 days) for patients who developed conjunctivitis. Median duration of yersiniosis symptoms did not differ significantly from case-patients who did not develop symptoms consistent with sequelae. The occurrence of symptoms of possible sequelae was not associated with antimicrobial treatment of the preceding *Y. enterocolitica* infection. Seventeen of 38 yersiniosis patients (45%) with at least one symptom of ReA, 5 of 11 (46%) yersiniosis patients with EN symptoms, and 8 of 16 (50%) patients with symptoms of conjunctivitis reported that they had consulted a physician because of the symptom(s).

Symptoms resembling possible sequelae of yersiniosis also occurred in the unexposed reference group, but the frequency was substantially lower than in yersiniosis patients (Table 4). Of the 39 persons in the reference group that reported symptoms similar to ReA, 6 (15%) had had diarrhea in the 7 days before completing the self-administered questionnaire, which may be an indication of a preceding gastrointestinal infection. None of them had consulted a physician because of the diarrhea. Eight of 37 persons in the reference group with symptoms resembling reactive arthritis (22%) visited a physician because of these symptoms.

## Discussion

In this population-based study, yersiniosis was associated with a considerable burden of illness. Symptoms of acute infection lasted for about 10 days, were associated with hospitalization in more than a quarter of yersiniosis patients, and led to absence from work for a median of 5 days in about half of the case-patients of working age or parents of sick children. Appendectomies and symptoms of probable ReA and EN were more frequently reported in yersiniosis patients than in persons without yersiniosis.

The proportion of patients with diarrhea was highest in children <5 years of age. Abdominal pain in the lower right quadrant was most predominant in children 5–14 years of age. This confirms results from previous studies that reported differences in clinical manifestation of *Y. enterocolitica* infection according to age [6,18-20]. In older children and adolescents infection is often characterized by terminal ileitis and mesenteric lymphadenitis with symptoms that may resemble appendicitis [20-22]. In our study, pain in the lower right abdomen was strongly associated with hospitalization and appendectomy. About 6% of case-patients aged ≥5 years underwent appendectomy associated with their *Y. enterocolitica* infection, whereas the background rate of appendectomies in the reference group was extremely low. Appendicitis may be a true complication of *Y. enterocolitica* infection in a small proportion of patients [21-23]. However, it is conceivable that at least some of the appendectomies were performed unnecessarily [21,22,24]. Sporadic cases of appendicitis are difficult to differentiate from cases of pseudoappendicitis due to *Yersinia* infection [22]. Terminal ileitis or mesenteric lymphadenitis caused by *Y. enterocolitica* infection should be kept in mind as a possible explanation for symptoms resembling appendicitis in older children and adults, and, provided that immediate surgery is not warranted, further diagnostic procedures including timely testing for *Y. enterocolitica* should be considered before appendectomy is performed.

A surprisingly high proportion of case-patients reported that the *Y. enterocolitica* infection was treated with antimicrobial drugs, especially case-patients ≥15 years of age. Treatment with antibiotics is recommended only for severe or complicated diarrheal infections, in particular for associated or suspected bacteremia, or in immunocompromised hosts [5,25,26]. There is no evidence that in the total 37% of case-patients in our study who received antimicrobials, the illness was so severe that this treatment was warranted. We did not find an association between presumed severity of illness and antimicrobial treatment, confirming results from studies which could not link duration of enteritis symptoms due to *Y. enterocolitica* infections to antimicrobial treatment [27-30]. Likewise, we did not find an association between antimicrobial treatment and the occurrence of probable postin-fectious complications. To reduce unnecessary use of antimicrobials, treatment should be confined to those patients for whom it is recommended in the respective clinical guidelines.

A substantial proportion (12%) of yersiniosis patients reported symptoms of probable ReA. This proportion was comparable to a population-based study that found new onset of rheumatologic symptoms in 14% of subjects with a preceding *Yersinia* infection [13]. In another study, 23% of patients reported new onset of joint pain within 4 weeks of a *Y. enterocolitica* infection [15]. After *Y. enterocolitica* or *Y. pseudotuberculosis* outbreaks, 9-12% of case-patients reported symptoms consistent with ReA [8,9,31]. Comparison of our study to other studies is hampered by the lack of a generally accepted definition of ReA [14,32] and by the small number of population-based studies on sequelae following sporadic *Y. enterocolitica* infections [13,15]. In our study, the frequency of occurrence of symptoms consistent with ReA was calculated based on patients’ self-reports of symptoms. This may result an overestimation of the frequency of ReA because case-patients may have reported musculoskeletal symptoms that were unrelated to the preceding *Y. enterocolitica* infection. However, a major strength of our population-based study is the inclusion of a reference group that allowed us to estimate the frequency of musculoskeletal symptoms similar to ReA in a population not affected by yersiniosis. Subtracting this background frequency yielded an overall proportion of about 7% of yersiniosis patients with symptoms attributable to probable “true” ReA. In persons aged ≥ 15 years, this proportion may be about 3-times as high. Selection of the reference group may have biased our results slightly. While the majority of persons in the reference group interviewed about occurrence of sequelae-like symptoms did not report any preceding gastrointestinal symptoms in the 7 days before they completed the original questionnaire, it is conceivable that some of them had been affected by a gastrointestinal infection or another bacterial infection in the time before they were interviewed that may have resulted in symptoms resembling the sequelae investigated here. If the preceding gastrointestinal infection had been caused by *Y. enterocolitica*, which we consider unlikely, this would have resulted in a small underestimation of the association between yersiniosis and symptoms of possible sequelae.

In our study as well as in others, an association of severity of acute illness and ReA was not found [9,15,33]. In contrast, the risk of ReA increased with severity of the preceding acute diarrheal illness caused by *Campylobacter*, *Salmonella*, or *Escherichia coli* infections [15,34-38]. Symptoms of EN were less common among yersiniosis patients (3%) compared with symptoms of ReA. The frequency of occurrence of EN symptoms in the reference group was very low, which resulted in a strong association of yersiniosis and EN. To our knowledge, the frequency of occurrence of EN after infection with *Y. enterocolitica* has not been investigated systematically in a large population-based study to date, although it has been known for decades that EN is a post-infectious complication of yersiniosis. Case-patients who developed symptoms of probable EN were younger overall than patients with probable ReA; the frequency of EN symptoms also increased with age.

Considering only the 3,000 *Y. enterocolitica* infections which are being notified in Germany annually, a minimum estimate of about 150 appendectomies and 500–600 cases of ReA and EN associated with *Y. enterocolitica* infections are expected to occur annually in Germany. However, only a small proportion of patients with gastrointestinal symptoms will consult a physician and not every gastrointestinal illness will be laboratory-diagnosed [39], a prerequisite for notification. Therefore, the true number of post-infectious complications is much higher. Since consumption of raw and undercooked pork is a known important risk factor for *Y. enterocolitica* infections, even in young children [1,3], the number of acute *Y. enterocolitica* infections as well as post-infectious complications may be reduced by continuously discouraging consumers from this risky eating habit.

## Conclusions

Acute yersiniosis as well as post-infectious complications cause considerable burden of illness. Symptoms lasted on average for about 10 days and the *Y. enterocolitica* infection was associated with hospitalization in more than a quarter of patients. A high proportion of yersiniosis patients was treated with antimicrobial drugs even though guidelines only recommend them for severe cases. This study provides evidence that yersiniosis is significantly associated with appendectomies and post-infectious complications such as ReA and EN. We found no evidence for an association of yersiniosis and conjunctivitis. Physicians should consider recent *Y. enterocolitica* infection as a differential diagnosis in patients with symptoms resembling appendicitis as well as in patients with symptoms of unclear arthritis.

## Competing interests

The authors declare that they have no competing interests.

## Authors’ contributions

BMR participated in the design of the study, acquired, analyzed, interpreted the data, and drafted the manuscript. DW and KS participated in the design of the study and critically revised the manuscript. MH participated in statistical analysis of the data and critically revised the manuscript. All authors read and approved the final manuscript.

## Pre-publication history

The pre-publication history for this paper can be accessed here:

http://www.biomedcentral.com/1471-2334/13/236/prepub
